# A personalized nutrition intervention advances meal timing and is associated with reduced postprandial lipid and inflammatory responses in night shift workers

**DOI:** 10.3389/fnut.2026.1912094

**Published:** 2026-10-08

**Authors:** Maaike van der Rhee, Tim J. van den Broek, Inês Chaves, Martijn E. T. Dollé, Linda W. M. van Kerkhof, Alex Burdorf, Heidi M. Lammers-Van der Holst, Johanneke E. Oosterman, Suzan Wopereis

**Affiliations:** 1 Department of Public Health, Erasmus MC, Rotterdam, Netherlands; 2 Research Group Microbiology and Systems Biology, Netherlands Organization for Applied Scientific Research (TNO), Leiden, Netherlands; 3 Department Molecular Genetics, Erasmus MC, Rotterdam, Netherlands; 4 The Netherlands Centre for Health Protection, National Institute for Public Health and the Environment (RIVM), Utrecht, Netherlands; 5 Department of Internal Medicine, Erasmus University Medical Centre, Rotterdam, Netherlands

**Keywords:** cardiovascular health, chrono-nutrition, inflammation, meal timing, metabolic health, night shift work, postprandial

## Abstract

**Background:**

Night work disrupts circadian rhythms and alters glucose homeostasis, increasing long term cardiometabolic risk. Although nutritional interventions show beneficial short-term effects under controlled conditions, evidence from real life settings in night shift workers remains limited.

**Methods:**

In a non-randomized controlled intervention study, healthy male night shift workers aged 18 to 60 years participated in a three-month personalized nutrition intervention following a one-month run-in period. Personalization was based on demographic characteristics combined with postprandial metabolic responses to a standardized mixed meal tolerance test. Advice focused on advancing the timing of energy intake, redistributing daily caloric and macronutrient intake to align with endogenous circadian rhythms during night shifts. Controls continued habitual dietary behavior. Food intake was assessed using a digital food diary, and diet quality by using the Dutch Healthy Eating Index at baseline, intervention start and intervention end. Postprandial cardiometabolic and inflammatory responses were assessed before and after intervention using a standardized mixed-meal challenge test, including glucose, insulin, cholesterol, triglycerides, and a panel of inflammatory markers. Participants wore a blinded continuous glucose monitoring device for 14 days at baseline, start and end of intervention.

**Results:**

In the nutrition intervention group (*n* = 22), the timing of total energy intake during night shifts advanced by 150 min at intervention start and 88 min at intervention end, compared with baseline. Total energy intake and diet quality remained unchanged, and no dietary changes were observed in controls. Postprandial total, LDL and HDL cholesterol levels responses were reduced following the intervention, while postprandial glucose, insulin and triglyceride responses did not change. Postprandial responses of several inflammatory markers, including IL-10, IL-8, E-selectin, ICAM-1 and TNF-α were lower after the intervention. Continuous glucose monitoring-derived metrics showed modest changes, suggesting alterations in glucose dynamics without improvements in overall glucose control.

**Conclusion:**

A three-month personalized nutrition intervention targeting meal timing and macronutrient distribution in night shift workers advanced the timing of energy intake and was associated with reduced postprandial lipid and inflammatory responses. These findings suggest that modifying meal timing and macronutrient distribution may support cardiometabolic health in real-world night work settings.

**Clinical trial registration:**

ClinicalTrials.gov, identifier NCT06147089.

## 1 Introduction

Night shift work is common, with 1 in 5 workers in the European Union engaged in night shifts (1). Working at night is associated with an increased risk of chronic diseases, such as cardiovascular disease (2), type 2 diabetes (3), or obesity (4, 5). Beyond disrupting sleep, night shift work substantially alters eating patterns as part of the occupational context, thereby disrupting normal eating-fasting patterns (6, 7). Night shift workers frequently consume food during biologically inappropriate hours, often constrained by work schedules, limited break times, and workplace food availability (8). Consequently, nutritional behavior during night shifts represents an important occupational factor that may contribute to adverse health outcomes.

Since metabolic processes in the human body are not optimized for eating at night, nocturnal food intake can increase postprandial blood glucose concentrations (9). These dysregulated acute post-meal responses are increasingly recognized as early and clinically relevant indicators of long-term cardiometabolic risk (10). Circadian misalignment has been shown to exacerbate these effects, with evidence demonstrating elevated glucose excursions and disrupted lipid metabolism (11, 12). Furthermore, night shift work has been linked to low-grade systemic inflammation, arising from circadian misalignment, sleep disruption, and behavioral changes such as altered eating patterns. This chronic inflammatory milieu may play a key role in the development of cardiometabolic disease, particularly through effects on glucose and lipid metabolism (13–15).

Recent studies have explored lifestyle-based strategies to mitigate the negative health effects of shift work (16, 17), with chrono-nutrition emerging as a promising approach. Chrono-nutrition aims to align food intake as much as possible with circadian rhythms to optimize metabolic health (18). While fasting during night shifts might reduce metabolic risks, complete fasting may be impractical for shift workers (16, 19). Therefore, pragmatic nutritional strategies that focus on adjusting meal timing and macronutrient distribution, rather than complete avoidance of intake, may offer a more feasible approach for night shift workers while still mitigating metabolic disruption.

However, much of the current research has been conducted in controlled laboratory settings, rather than in real-world workplace settings. In addition, while postprandial glucose responses in shift workers have been relatively well studied (i.e., (20)), data on postprandial inflammatory responses remain underexplored, and comprehensive phenotyping using standardized mixed meal challenge tests is rare. As a result, the combined cardiometabolic and inflammatory consequences of nocturnal eating during habitual night shift work remain insufficiently understood.

To address this, the current study evaluates the cardiometabolic and inflammatory impact of personalized guidelines for meal timing and macronutrient distribution in a real-world night shift workplace setting. The primary aim of the intervention was to promote circadian alignment and minimize cardiometabolic disruption. Given the substantial individual variability in responses to shift work and meal timing preferences, a personalized approach was used to enhance applicability and effectiveness (21–23). Such variability may be particularly evident in postprandial metabolic responses to nocturnal meals, suggesting that individualized nutritional guidance informed by objective metabolic measurements could be of added value. To evaluate the cardiometabolic and inflammatory impact of these guidelines under real-life conditions, the study incorporated standardized assessment of postprandial responses to a mixed-meal challenge test alongside continuous monitoring of glucose dynamics during habitual night shift work.

This study is part of a larger intervention study (extensively described in (24)), in which multiple determinants of cardiometabolic health were assessed in response to a personalized nutrition or sleep intervention. For the current study, we assessed the effect of meal timing and macronutrient distribution on postprandial inflammatory responses and glucose dynamics. Data from the sleep intervention and control conditions where available, are presented for context, but no direct comparisons between intervention strategies were performed. We hypothesized that a personalized adjustment of meal timing and macronutrient distribution would shift the timing of energy and macronutrient intake towards earlier hours, while maintaining total caloric intake. This shift was expected to be associated with improvements in postprandial cardiometabolic and inflammatory biomarker responses following 12 weeks of intervention.

## 2 Materials and methods

### 2.1 Study design

In this 3-months, non-randomized, controlled intervention, healthy male night shift workers aged 18 to 60 years self-selected into a nutrition intervention (*n* = 22), sleep intervention (*n* = 25) or control group (*n* = 10), to support autonomy and intervention adherence. The full study protocol has been described in detail elsewhere (24). For the present study, data were collected at baseline (run-in period), during the first weeks of the intervention (start of intervention), and during the final weeks of the 3-months intervention (end of intervention). Data collection took place from 2023 until 2025, with continuous participant enrollment during the first year. The study was approved by the Medical Ethical Review Committee of Erasmus University Medical Center (NL82649) and registered at ClinicalTrials.gov (NCT06147089).

### 2.2 Recruitment and study population

Participants were recruited from a single company in the Port of Rotterdam, the Netherlands. They were healthy male shift workers, whose work primarily involved short bouts of heavy lifting, associated with docking operations. Eligibility criteria included an age range of 18–60 years, a minimum of 20 working hours per week, and at least one year of night shift experience. Exclusion criteria were a medical history of cardiovascular, bleeding, or inflammatory disease, the use of sleep medication, and habitual alcohol intake exceeding 21 units per week.

Participants all worked 12-h rotating shifts (06:00–18:00; 18:00–06:00), typically consisting of 7 day shifts and seven night shifts per month. Work schedules generally comprised two or three consecutive workdays followed by two rest days. Written informed consent was obtained from all participants prior to study participation.

### 2.3 Nutrition intervention

The nutrition intervention consisted of personalized dietary advice tailored to each participant’s cardiometabolic health status and dietary preferences. Between runin start and intervention start, participants received one-on-one feedback on their health data and guidance on adapting their dietary behavior. Through goal setting, they formulated individual goals for dietary behavior change. The nutritional advice was developed in four steps (Figure 1), covering calorie distribution across the 24-h period, macronutrient distribution based on the literature and mixed-meal tolerance test results, continuous glucose monitoring (CGM)-informed food choices, and consideration of personal preferences.

**Figure 1:**
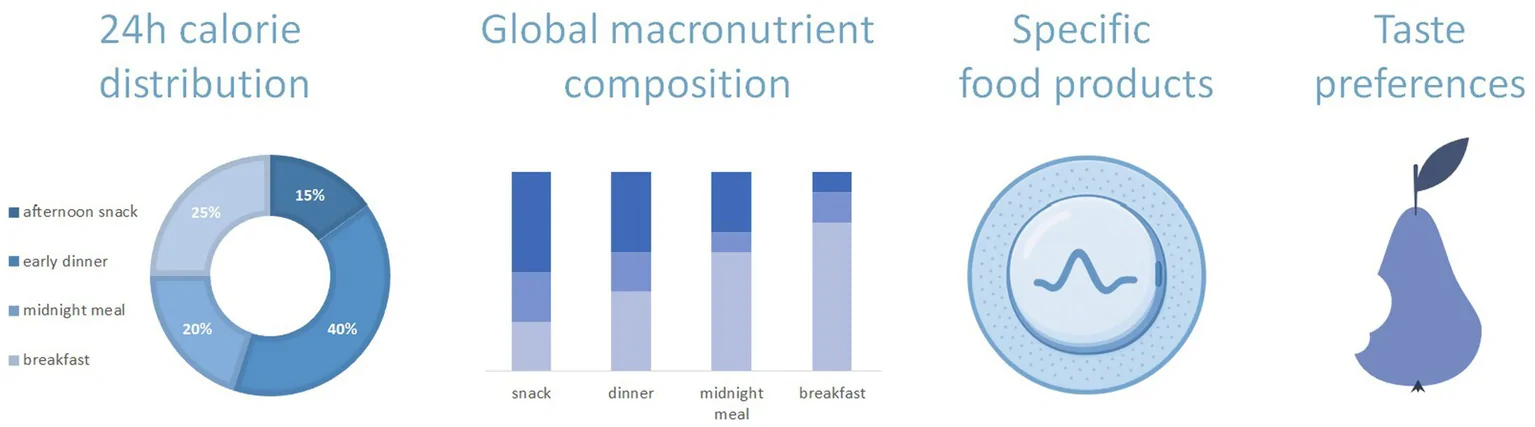
Personalized nutritional advice based on a four-step approach. Personalized nutritional advice was developed in four steps: (1) distribution of energy-intake across the 24-h period before, during, and after night shifts, tailored to individual needs; (2) macronutrient distribution over the four-meals based on literature (16) and individual mixed meal challenge test results; light blue: carbohydrate; mid-blue: protein, dark blue: fat (3) food product choices informed by continuous glucose monitoring; and (4) integration of individual preferences.

The four-meal structure (i.e., a light midnight meal, a post-night shift breakfast, an afternoon snack, and dinner) is based on approaches previously described by Gupta and colleagues (16, 25), who reported beneficial effects of this meal structure on sleepiness and cognitive performance under controlled laboratory conditions. An example of such personalized nutrition advice for one of the participants is provided in Supplementary Information S1.

Participants received a 24 h calorie distribution for night shifts based on estimated individual energy needs, together with a macronutrient distribution across the 4 -meal structure that could be further tailored to their baseline cardiometabolic health status, food products they were advised to avoid, and recipe suggestions consistent with the advice.

The personalized nutritional advice was intended for the days with night shifts and the morning after. During free days and day shifts, participants could follow their habitual eating schedule. Participants were instructed to distribute their caloric intake over four meals: a light midnight meal (20% of total intake, between 0:00 and 2:00), a post-shift breakfast (25%, between 6:00 and 8:00), an afternoon snack (15%, between waking up – 16:00), and dinner (40%, between 18:00 and 20:00) (16, 24). Macronutrient composition was aligned with circadian rhythms in glucose tolerance and insulin sensitivity, with relatively a higher proportion of carbohydrates in the morning and a higher proportion of fat in the evening (16). The nutritional advice specified the macronutrient distribution for each eating occasion as follows: breakfast 75% carbohydrates (CHO), 15% protein (PRO) and 10% fat (F); afternoon snack 25% CHO, 25% PRO and 50% F; early dinner 45% CHO, 20% PRO, and 35% F; and light midnight meal 55% CHO, 15% PRO and 30% F. Total amount of calories was tailored to each participant’s individual energy needs equaling estimated energy expenditure, calculated based on baseline runin start age, gender, weight, and activity level (26).

Further personalization was informed by results from a mixed-meal challenge test performed on a non-workday (PhenFlex Test, FoodPilot, Belgium). Based on the postprandial glucose and insulin values, tissue-specific insulin resistance phenotypes could be distinguished (27). Participants with muscle insulin resistance (MIR) were advised to follow a diet enriched in monounsaturated fats (28). Those with liver insulin resistance (LIR) received recommendations for a low-fat, high-protein, high-fiber diet (28). Participants with elevated circulating lipids were advised to follow a low-saturated fat, high-fiber diet.

### 2.4 Control group and sleep intervention

The sleep intervention consisted of personalized, evidence-based recommendations targeting individual sleep issues identified at baseline. Personalization was guided by a combination of objective and subjective sleep assessments, which were used to identify the most prominent sleep problems for each participant. The intervention aimed to improve sleep timing, duration, or quality, while maintaining habitual lifestyle behaviors related to nutrition and physical activity. The control group was instructed to maintain their habitual lifestyle, including sleep and dietary behaviours, while participating in study visits, online assessments outside the visits, and a multiple 14-day monitoring periods covering shifts (including continuous glucose monitoring). Further details on the development, categorization of sleep issues, and implementation of the personalized sleep intervention are described elsewhere (29).

### 2.5 Data collection and measurements

#### 2.5.1 Assessment of dietary intake

Food intake was tracked using short-term food logs, which participants filled in via a smart phone application (TNO, Leiden, The Netherlands) during 2 nights shifts and 1 free day for each measurement period (runin start, intervention start, intervention end). In this app, timestamps for each food and drink consumption were tracked, as well as calories and macronutrients per consumption. Diet quality was evaluated at runin start and intervention end using the Eetscore, which calculates the Dutch Healthy Diet Index (DHDI), a measure of diet quality reflecting adherence to the 2015 Dutch dietary guidelines. The DHDI score ranges from 0 to 160, with higher scores reflecting better diet quality (30).

#### 2.5.2 PhenFlex challenge test and biomarker measurements

The PhenFlex Test consists of a high-caloric drink (400 mL, 950 kcal) containing 75 g dextrose, 60 g palm oleine, 20 g casein-based protein, and 0.5 g artificial vanilla aroma (31). On the evening prior to testing, participants were instructed to consume a low-fat meal, abstain from alcohol and refrain from vigorous exercise. Participants were instructed to consume the PhenFlex drink within 5 to 10 min. Plasma samples were collected via a venous cannula in the forearm under overnight fasting conditions (*t = 0*), and at 30, 60, 120, and 240 min after consumption at baseline runin start and at the end of the intervention.

From these repeated blood samples, fasting and postprandial concentrations of glucose, insulin, triglycerides, LDL- and HDL-cholesterol were measured in the clinical diagnostic lab of the Erasmus Medical Center Rotterdam. Inflammatory markers interleukin (IL)-6, Il-8, Il-10, tumor necrosis factor (TNF)-α were measured by using multiplex immunoassays (Multiplex Panel Human; Meso Scale Discovery). The levels of serum amyloid A (SAA), E-selectin, P-selectin, soluble intercellular adhesion molecule (sICAM)-1, total plasminogen activator inhibitor (PAI)-1, and soluble vascular adhesion molecule (sVCAM)-1 were determined by ELISA (R&D Systems, Duoset). Of note, the PhenFlex test was performed exclusively in the nutrition intervention group.

#### 2.5.3 Continuous glucose monitoring

All participants wore a blinded continuous glucose monitoring device (CGM, FreeStyle Libre Pro; Abbott) during three measurement periods (runin start, intervention start, intervention end). Each measurement period lasted 14 days and covered night shifts, early shifts, and rest days. Interstitial glucose concentrations were recorded every minute. Based on the collected CGM data five glycemic outcomes were derived (32, 33). Time in range (TIR) was defined as the percentage time within the target glucose range (3.9–10.0 mmol/L). The Glucose Management Index (GMI) was calculated to estimate overall mean glucose exposure. The High Blood Glucose Index (HBGI) was used to quantify the frequency and severity of hyperglycemic excursions using a risk-weighted transformation of glucose values. The Mean Glycemic Excursion (MGE) was calculated as the mean of glucose values lying more than one standard deviation above or below the mean glucose level. Finally, the Interday Coefficient of Variation (CV) was computed as the standard deviation of daily mean glucose divided by the mean daily glucose, multiplied by 100, to assess day-to-day variability in glucose levels.

TIR, GMI, HBGI, and MGE were calculated separately for days that included night shifts and for days that included day shifts or free days. Interday-CV was calculated across the full monitoring period to capture overall day-to-day glycemic variability. This outcome is particularly relevant in night shift workers, as alternating work schedules may contribute to fluctuations in glucose regulation. By including day shifts and rest days in addition to night shifts, we were able to evaluate whether optimization of meal timing and macronutrient distribution was associated with reduced glycemic variability across different shift types, indicative of a more stable metabolic profile.

### 2.6 Statistical analysis

To evaluate whether nutritional behavior changed over time, we analyzed repeated measurements using generalized estimating equations (GEE), with participant as the clustering variable and an exchangeable working correlation structure. We focused on the weighted median timing of total energy intake during night shifts and the weighted median timing of carbohydrate, protein, and fat intake. The weighted median was calculated separately for each participant, night-shift day, measurement occasion, and nutrient variable. For each eating occasion, clock time was converted to seconds after midnight and weighted according to the amount of energy consumed at that occasion. Eating occasions were ordered by clock time, and the weighted median was defined as the time at which the cumulative energy intake reached or exceeded 50% of the total energy intake for that day. When cumulative intake was exactly 50% at an eating occasion, the midpoint between the time of that occasion and the subsequent eating occasion was used. Thus, larger meals contributed proportionally more to the determination of the median intake time than smaller meals or snacks. For macronutrient-specific analyses, energy intake was calculated as 4 kcal/g for carbohydrate and protein and 9 kcal/g for fat. The resulting day-level weighted median times were used as repeated outcomes in the GEE models to assess within-group changes over time in the nutrition, sleep, and control groups.

To assess whether postprandial biomarker responses changed over time, repeated biomarker measurements during the mixed-meal challenge were analyzed in the nutrition group using GEE models with participant as the clustering variable and an exchangeable working correlation structure. For each biomarker separately, the model included timepoint (0, 30, 60, 120, and 240 min), occasion (runin start and intervention end), and the timepoint × occasion interaction. This interaction term was used to test whether the postprandial response profile differed between runin start and intervention end. The biomarker models were fitted on log-transformed concentrations. Thus, one omnibus interaction test was performed per biomarker. Timepoint-specific contrasts consisted of one intervention end-versus-runin start comparison at each timepoint and were used to describe where differences in the response profile occurred.

CGM-derived summary metrics were analyzed separately for each outcome using GEE models with participant as the clustering variable. For TIR, GMI, HBGI, and MGE, models included group, occasion, shift type, and their interactions. Interday-CV was analyzed separately across the full monitoring period using group, occasion, and their interaction. The CGM metric models were fitted on square-root transformed metric values. Diet quality, assessed by using the Dutch Healthy Diet Index (DHDI), was analyzed using GEE models with participant as the clustering variable and fixed effects for group, occasion, and their interaction. DHDI scores were square-root transformed prior to analysis.

Estimated marginal means and *post hoc* within-group and between-group contrasts were obtained using the emmeans package. Pairwise contrasts were evaluated without adjustment for multiple comparisons. Analyses were performed in R version 4.5.1 (34) using the packages geepack version 1.3.13 (35) and emmeans version 1.11.2–8 (36). A two-tailed *p*-value < 0.05 was considered statistically significant.

## 3 Results

### 3.1 Demographics of study population

At baseline, the mean age was higher in the nutrition intervention group (38 yr. ± 10) compared to the sleep intervention (36 yr. ± 7) and control groups (31 yr. ± 4) (Table 1). The control group displayed a more favorable cardiometabolic profile, whereas the nutrition intervention group had higher proportions of participants with a BMI above 25 kg/m^2^, waist circumference, waist-to-hip ratio, fasting glucose, triglycerides, and total cholesterol levels (Table 1).

**Table 1:** Descriptives of percentage of participants with increased body mass index (BMI), waist circumference, waist-hip ratio, fasting glucose, fasting triglycerides, total cholesterol.

Variable - cut-off value	Intervention group nutrition	Intervention group sleep	Control group
Age (yr ± SD)	38 (±10)	36 (±7)	31 (±4)
% BMI > 25 kg/m^2^	81	50	38
% Waist circumference > 94 cm	57	29	0
% Waist-hip ratio > 0.9	76	54	38
% Fasting glucose ≥5.6 mmol/L	33	29	13
% Fasting triglycerides ≥ 1.7 mmol/L	10	8	0
% Total cholesterol ≥ 5 mmol/L	68	46	13

### 3.2 Effect of nutrition intervention on food intake and CGM metrics

#### 3.2.1 Advanced food-intake timing following nutrition intervention

In the nutrition intervention, the weighted median timing of total energy intake during night shifts shifted from 22:20 at baseline to 19:50 h at the start of the intervention, corresponding to a 150 min earlier timing (Figure 2, Table 2). At the end of the intervention, this shift remained 88 min earlier compared to baseline. Specifically, carbohydrate intake shifted 125 min earlier at the start of the intervention and 79 min earlier at the end of the intervention, while fat intake shifted 125 min earlier at the start and 84 min earlier at the end of intervention, compared to baseline. Protein intake showed a similar pattern, with a 129 min earlier timing at the start and 84 min earlier timing at the end of the intervention. No significant changes in the timing of food intake were observed in the control or sleep intervention groups (Figure 2, Supplementary Table 1).

**Figure 2:**
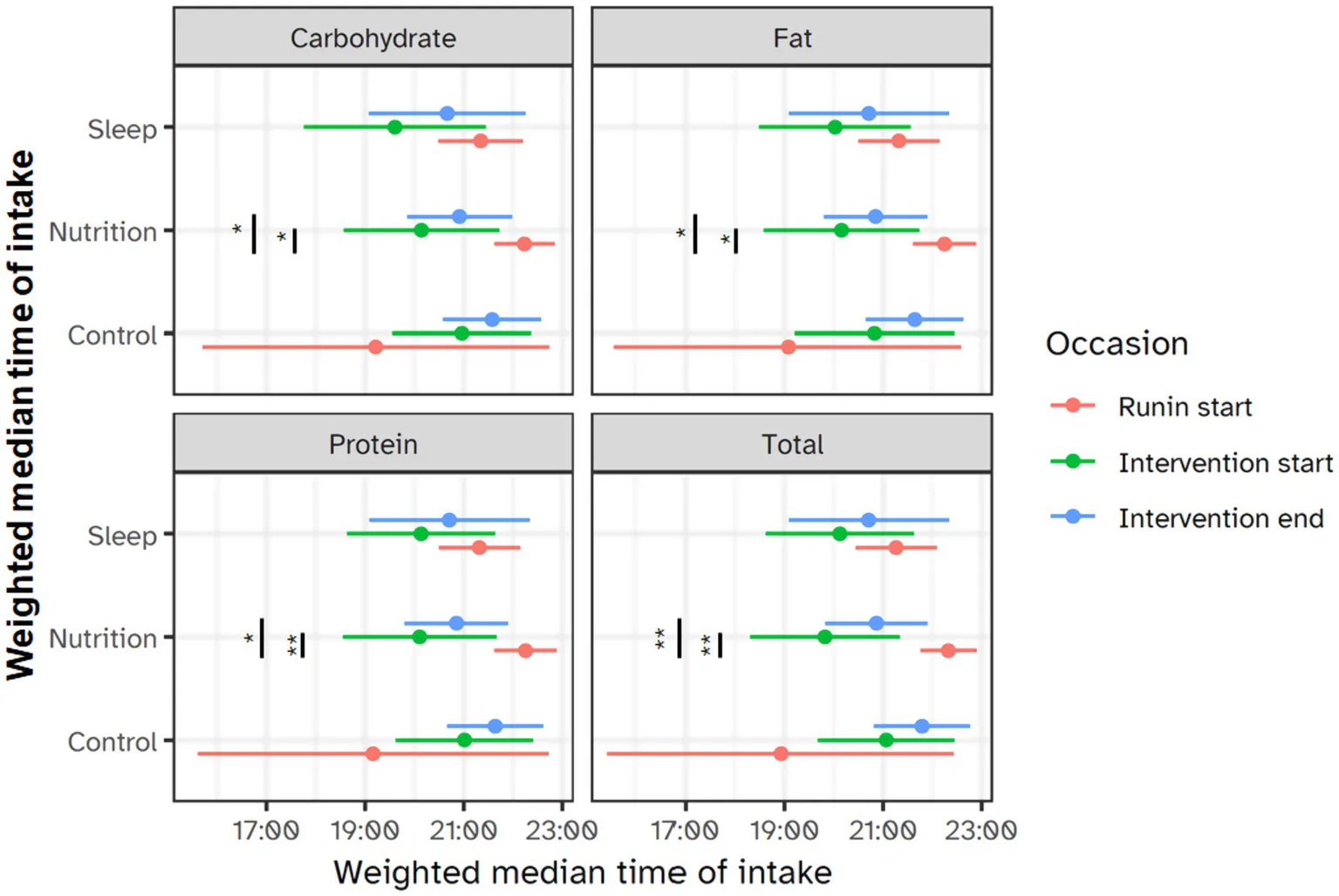
The weighted median time of energy intake for the macronutrients (carbohydrate, fat, protein) and total caloric intake. Points represent the median, whiskers the 95% Confidence Interval; *p ≤ 0.05, **p ≤ 0.01, ***p ≤ 0.001 based on a within group comparison. X-axis: clock time.

**Table 2:** Changes in the timing of food intake in the nutrition group for total caloric intake and macronutrients.

Variable	Start intervention vs Run-in estimate [lower CI, Upper CI]	End intervention vs Run-in estimate [lower CI, Upper CI]
Total	**−150 [−246, −55]**	**−88 [−146, −29]**
Carbohydrate	**−125 [−220, −30]**	**−79 [−142, −16]**
Fat	**−125 [−224, −27]**	**−84 [−149, −19]**
Protein	**−129 [−226, −32]**	**−84 [−149, −20]**

Estimate represents the changes of timing in minutes, with a negative value indicating earlier timing. Bolded value indicates statistical significance.

#### 3.2.2 No changes in total energy intake and diet quality following nutrition intervention

At baseline, mean daily energy intake was 1965 ± 156 kcal in the nutrition group, versus 1703 ± 302 kcal in the control group, and 2,159 ± 618 kcal in the sleep group. Total energy intake did not change over time in any of the groups (Supplementary Table 2). Baseline Dutch Healthy Diet Index (DHDI) scores were 91.9 ± 5.30 in the nutrition group, 85 ± 4.28 in the control group, and 85.4 ± 3.82 in the sleep group, respectively. No significant changes over time were observed for the nutrition group (*p* = 0.59), nor for the control group (*p* = 0.30). In contrast, the sleep group showed a significant increase in DHDI score at the end of the intervention (7.44 points ± 2.28, *p* = 0.001) as compared to baseline values.

#### 3.2.3 Effects of interventions on interstitial glucose values

Figure 3 shows the CGM-derived metrics Glucose Management Index (GMI), High Blood Glucose Index (HBGI), Mean Glycemic Excursion (MGE), and Time-In-Range (TIR) over time, stratified by days with night shifts and days with days shifts. Within group comparisons in the nutrition intervention group showed changes in several CGM-derived metrics over the course of the intervention. GMI increased at the end of the intervention compared to baseline and start of intervention on days with day shifts (estimate = 0.15%, *p* = 0.014 and estimate = 0.09%. *p* = 0.011 respectively) and compared to baseline on days with night shifts (estimate = 0.14%, *p* = 0.018). Similarly, HBGI increased at the end of the intervention compared to start of intervention on day shift days (estimate = 0.07, *p* = 0.009) and compared to baseline on night shift days (estimate = 0.09, *p* = 0.013). MGE also increased in the nutrition intervention group at the end of the intervention compared to baseline and intervention start, both on days with day shifts (estimate = 0.34 mmol/L, *p* = 0.043 and estimate = 0.31 mmol/L, *p* = 0.003 respectively) and compared to baseline on days with night shifts (estimate = 0.49 mmol/L, *p* = 0.003). In contrast, TIR increased during days with night shifts, when comparing the end of the intervention with the start of intervention (+1.13%, *p* = 0.041).

**Figure 3:**
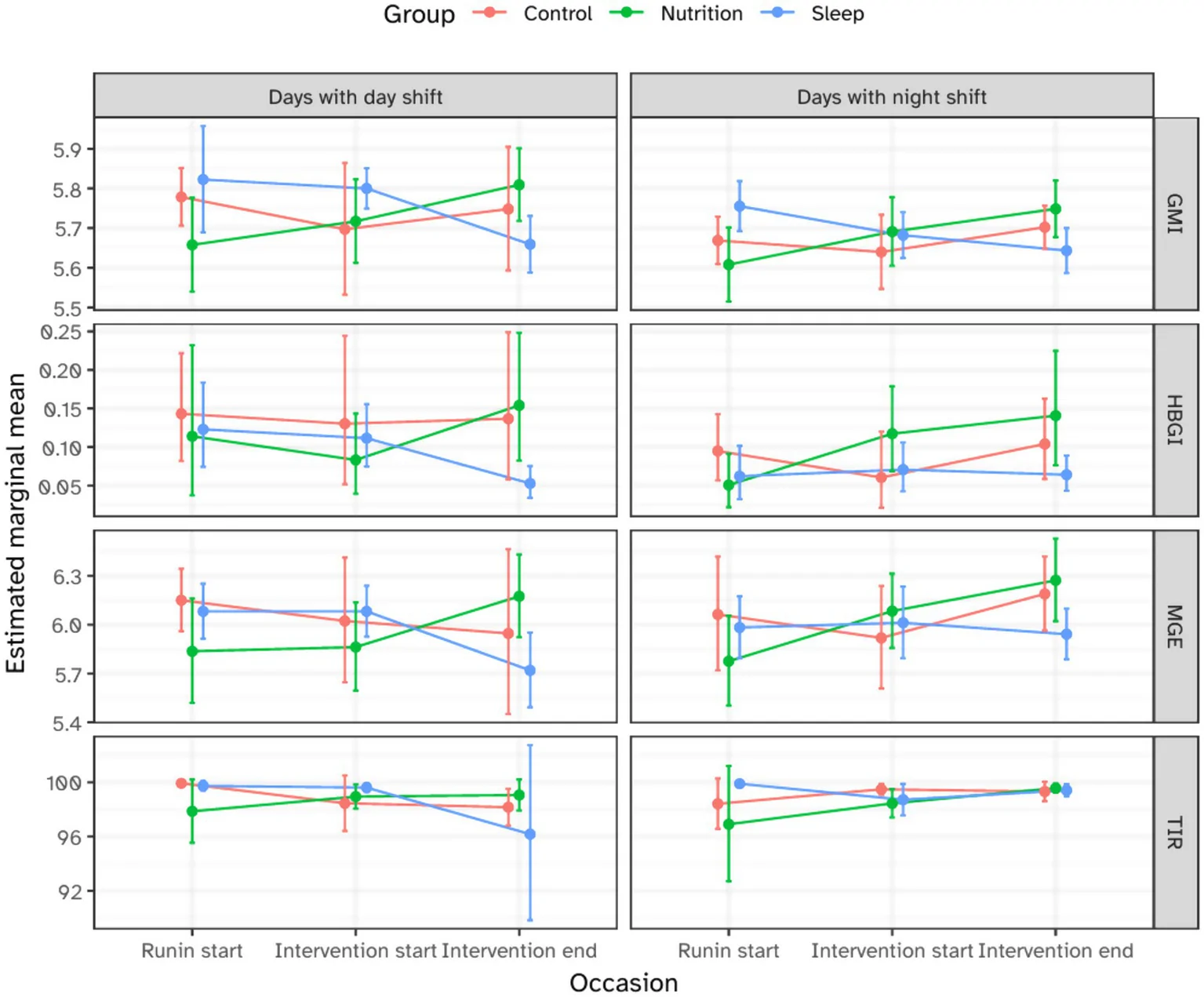
CGM-derived metric for all groups over time, stratified by days with nights shifts and days with day shifts. Points represent the median, whiskers the 95% confidence Interval. GMI, glucose management index (in percentage); HBGI, high blood glucose index; MGE, mean glucose excursions (in mmol/L); TIR, time in range (in fraction of daylength).

Data from the sleep intervention and control conditions are reported based on within-group comparisons (Figure 2). In the sleep intervention group, GMI decreased between baseline and the end of the intervention on both day shift and night shift days, while HBGI and MGE decreased during day shift days only. TIR decreased in the sleep intervention group when contrasting intervention-start and -end with baseline. No significant changes were observed in the control group for GMI, HBGI, MGE, or TIR. Additionally, no significant changes in interday-CV were observed in any group (Figure 4).

**Figure 4:**
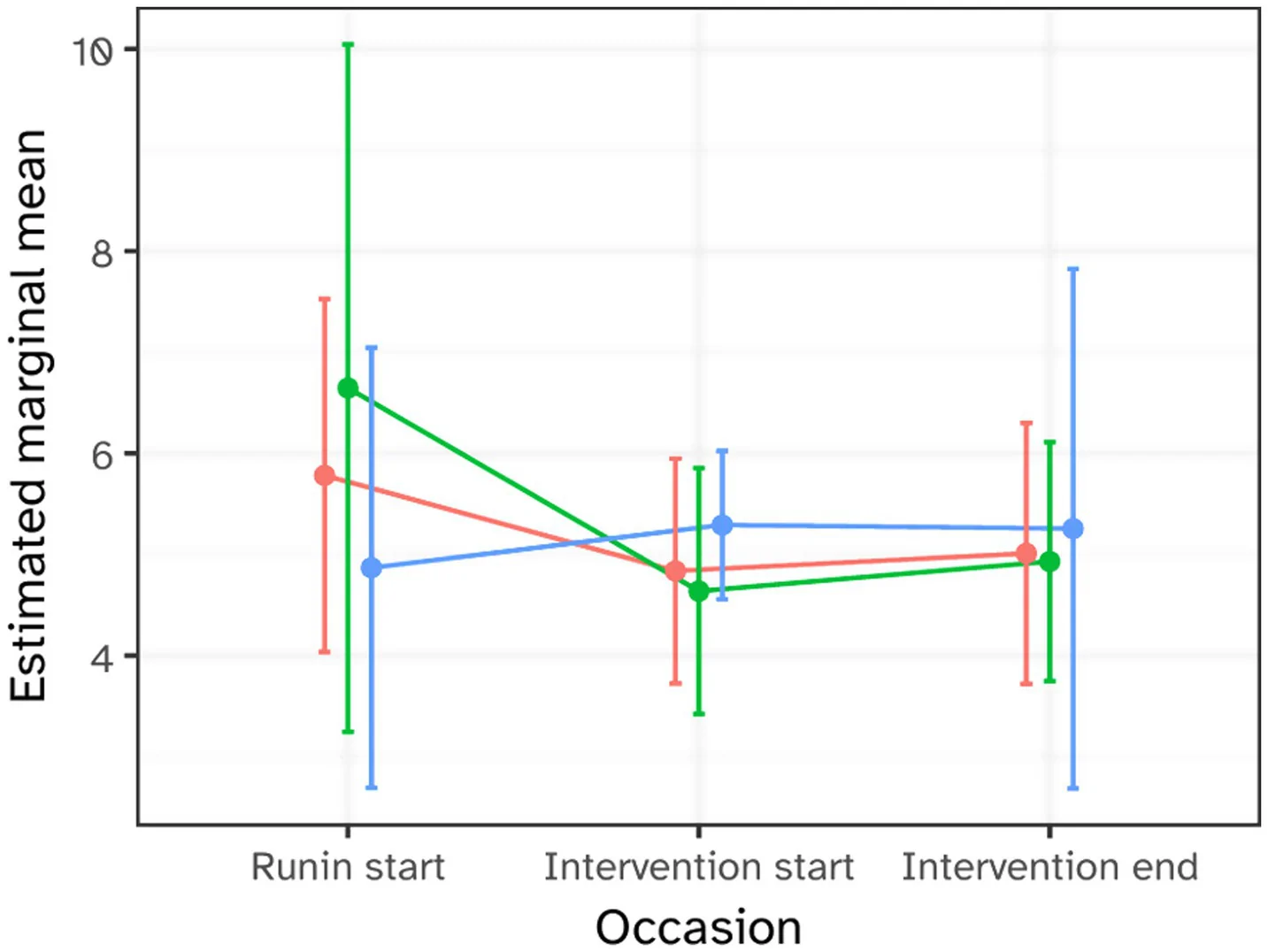
CGM-derived Interday Coefficient of Variation (in percentage) for all groups over time. Points represent the median, whiskers the 95% confidence Interval.

### 3.3 Effects of the nutrition intervention on postprandial biomarker responses

#### 3.3.1 Postprandial lipid responses to the mixed-meal challenge

Figure 5 shows the postprandial responses to the mixed-meal tolerance test in the nutrition group. Significant timepoint x occasion interactions were observed for total cholesterol, LDL cholesterol and HDL cholesterol (*p* = 0.025, *p* = 0.013 and p = <0.001 respectively), indicating differences in postprandial response patterns over time. *Post hoc* analyses revealed no significant pairwise differences between timepoints. Visual inspection suggested a lower postprandial response curve particularly for LDL-cholesterol (Figure 4). No significant effects were found for postprandial responses to the mixed meal tolerance test for cholesterol-HDL ratio, glucose, insulin and triglycerides.

**Figure 5:**
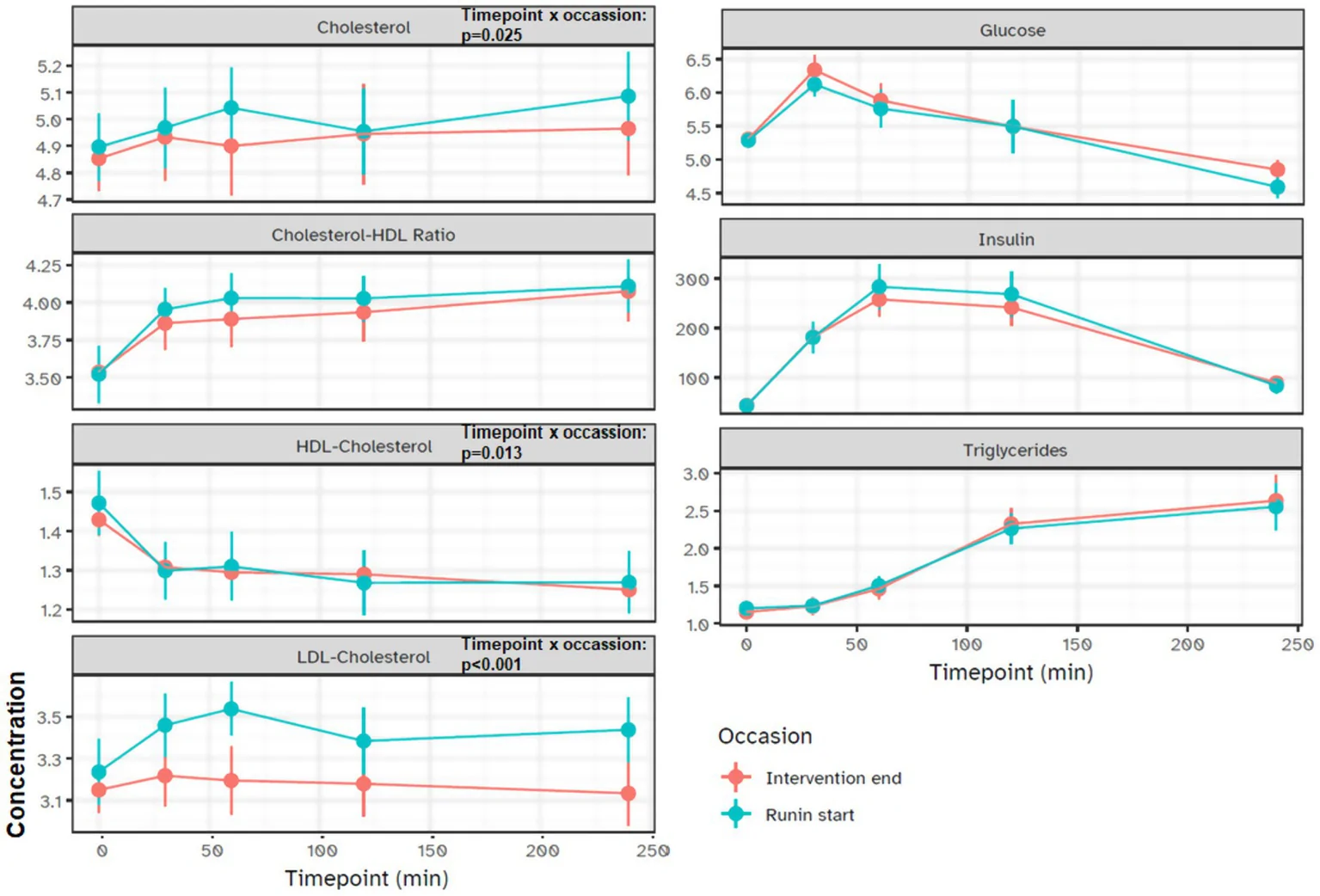
Cardiometabolic Postprandial responses to the Mixed-Meal Tolerance Test at Baseline and End of Intervention. Points represent the median, whiskers the 95% confidence Interval. Units for the biomarker concentrations were as follows: cholesterol (mmol/L), insulin (pmol/L), glucose (mmol/L), and triglycerides (mmol/L). The blue line represents baseline values, and the red line represents end-of-intervention values. *p ≤ 0.05, **p ≤ 0.01, ***p ≤ 0.001 based on *post-hoc* tests.

#### 3.3.2 Postprandial inflammatory responses to the mixed-meal challenge

Postprandial inflammatory markers, including SAA (*p* = 0.018), TNF-α (p = <0.0001), VCAM-1 (*p* = 0.0025), ICAM-1 (*p* = 0.017), and IL-10 (*p* = 0.036) showed significant timepoint x occasion interactions, indicating differences in postprandial response patterns over time. *Post hoc* analyses revealed limited significant pairwise differences between timepoints. Visual inspection of the response curves (Figure 6) suggested a tendency towards lower postprandial concentrations at the end of the intervention compared to baseline. Inflammatory marker IL-8 showed a significant main effect of occasion (*p* = 0.017), with postprandial concentrations differing between the end of the intervention compared to baseline, while IL-6 showed a tendency for timepoint x occasion (*p* = 0.086). The postprandial response of inflammatory markers P-selectin, Total PAI-I, and E-Selectin did not significantly change as a result of the nutritional intervention (Figure 6).

**Figure 6:**
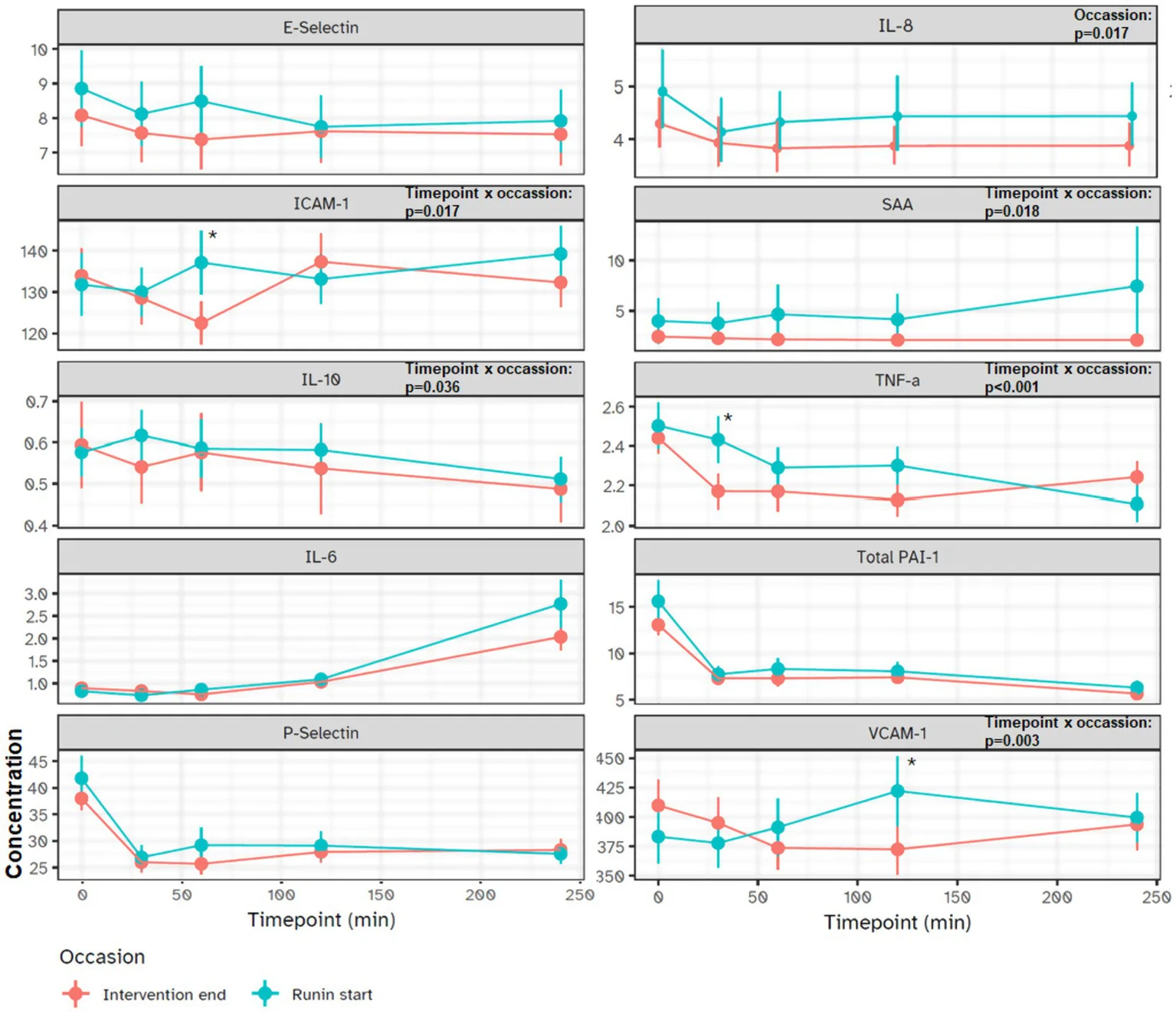
Inflammatory post-prandial responses to the mixed-meal tolerance test at run-in and end intervention. SAA, serum amyloid A; TNF-α, tumour necrosis factor-α; PAI-1, plasminogen activator inhibitor-1; VCAM-1, vascular adhesion molecule-1; ICAM-1, intercellular adhesion molecule-1; IL, interleukin. Points represent the median, whiskers the 95% confidence Interval. Units for the biomarker concentrations are in P-Selectin (ng/mL), SAA (μg/mL), TNF-a (pg/mL), Total PAI-1 (ng/mL), VCAM-1 (ng/mL), E-Selectin (ng/mL), ICAM-1 (ng/mL), IL-10 (pg/mL), IL-6 (pg/mL), IL-8 (pg/mL). *p ≤ 0.05, **p ≤ 0.01, ***p ≤ 0.001 based on *post-hoc* tests.

## 4 Discussion

This study assessed the effects of a personalized nutrition intervention focused on meal timing and macronutrient distribution in night shift workers. To our knowledge, this is the first real-life nutrition intervention that focused on postprandial inflammatory responses and glucose dynamics to assess the health impact of chrono-nutrition in shift workers.

We found that a 3-month personalized nutrition intervention advanced the timing of food intake which was associated with more favorable postprandial lipid and inflammatory response patterns. In addition, modest changes in CGM-derived metrics suggested alterations in glucose dynamics across both day and night shifts, without clear evidence for improved glucose-related metabolic outcomes. It is important to note that the nutritional intervention was relatively modest in scope, involving only a change in the timing of energy intake and macronutrient distribution on days with night shifts, which occurred on average 3 days per week. Total energy intake and overall diet quality were not altered. Despite this limited intervention intensity, changes in postprandial lipid and inflammatory responses were observed, suggesting that even relatively small, targeted modifications in meal timing during night shift work may contribute to more favorable postprandial lipid and inflammatory response patterns.

Our findings demonstrate that personalized nutrition advice targeting meal timing and macronutrient distribution during night shifts is feasible in a real-life shift work setting and can meaningfully advance the timing of food intake (approximately 1.5–2.5 h for total energy intake). This is in line with evidence from earlier studies that an early meal schedule has better metabolic health outcomes compared to a late schedule (37). An earlier publication from this study reported high adherence to the nutrition advice at mid-intervention (94%) and at end of intervention (95%), which remained relatively high at 8 months follow-up (73%), with participants reporting applying the advice during at least half of their night shifts (29). These adherence results further support the real-world feasibility of this nutritional intervention.

Within the nutrition intervention group, we observed the reduction in postprandial cholesterol response after a standardized high-caloric mixed-meal challenge test, including total, LDL, and HDL cholesterol. The cholesterol-HDL ratio remained unchanged postprandially, suggesting that these changes in postprandial cholesterol responses did not affect lipid profile quality. To our knowledge, limited research has examined the effects of meal timing on cholesterol levels in night shift workers. A small study by Leung and colleagues asked night shift workers with abdominal obesity, who habitually consumed food during night shifts, to adhere to a shorter overnight fasting window (01:00 h–06:00 h). This intervention shows no effect on cholesterol levels (38). Our results suggest that meal timing modifications may influence postprandial rather than fasting lipid responses, even in the absence of changes in energy intake or diet quality. This effect was observed in the absence of weight loss, indicating that meal timing itself may play a role in modulating postprandial cholesterol metabolism. Furthermore, evidence suggests that the relationship between shift work and cholesterol is complex and may, in part, be influenced by the distribution and quantity of food consumed during night shifts. For instance, a small cross-sectional study indicated that among night shift nurses, greater food intake during night shifts was associated with higher fasting cholesterol levels (39). While these findings are based on fasting lipid levels and therefore not directly comparable to postprandial responses, they may point to similar underlying mechanisms, suggesting that reducing food intake during the night may contribute to lower postprandial cholesterol responses. This pattern may reflect changes in lipoprotein metabolism, including reduced production and/or enhanced clearance of circulating lipoproteins during the postprandial phase.

The observed changes in postprandial inflammatory markers suggest an overall reduced inflammatory response within the nutrition intervention group. While these findings may be consistent with a more favorable acute inflammatory profile, they should be interpreted cautiously given the absence of a concurrent control comparison. Markers reflecting the acute phase inflammatory response, including TNF-α, IL-6, IL-8, and IL-10, showed a consistent tendency towards lower postprandial response patterns, in line with previous findings that dietary interventions, such as whole-grain wheat consumption, that have been shown to modulate this acute phase postprandial inflammatory response (40, 41). Our study observed a similar pattern, where postprandial responses of this set of biomarkers shifted towards a phenotype resembling that of younger and healthier individuals, characterized by lower postprandial inflammatory responses (41). In addition, reduced postprandial response patterns were observed in markers related to vascular and endothelial inflammation, including ICAM-1, VCAM-1, and SAA. This pattern partially overlaps with observations from energy restriction studies, where postprandial improvements were mainly observed in endothelial markers (ICAM-1, VCAM-1, SAA, PAI-1 and E- and P-selectin), with no effects of postprandial acute phase inflammatory response (42). Similar to our findings, these energy restriction studies also reported reduced postprandial responses of these endothelial markers, although the underlying intervention differed in nature. Together, these findings suggest that the present intervention may have influenced both cytokine-driven and vascular-related postprandial inflammatory processes, although not uniformly across all markers. Aligning food intake with circadian rhythms may reduce metabolic stress during night shifts by improving the alternation between anabolic and catabolic phases, thereby attenuating inflammatory responses. Evidence from non-shift work populations indicates that time-restricted eating may modestly reduce pro-inflammatory cytokines, including TNF-α, although these effects are often observed in conjunction with weight loss (43). A study among 24-h shift workers showed that 12 weeks of time-restricted eating improved cardiometabolic risk factors, including reductions in body weight, and reduced C-reactive protein in individuals with elevated baseline inflammation (44). Although chrono-nutrition is increasingly recognized as a promising strategy to mitigate the metabolic consequences of shift work, evidence on its effects on inflammatory outcomes in night shift workers remains scarce. Most intervention studies in shift workers have primarily focused on cardiometabolic endpoints, with limited or no assessment of inflammatory markers. To our knowledge, this is the first study to evaluate postprandial inflammatory biomarker responses under real-world night shift conditions.

In contrast to the improvements observed in cholesterol and inflammatory biomarkers, we found no significant postprandial changes in glucose and insulin levels following the nutritional intervention. This was unexpected, as previous studies indicate that eating during the biological night impairs glucose handling and overall glucose tolerance under simulated night work conditions, whereas interventions that reduced or shifted nocturnal food intake showed improved glucose metabolism. For example, Centofanti et al. demonstrated that consuming a large snack around midnight increased the post breakfast interstitial glucose response after a night shift compared with a smaller snack, suggesting that not only timing but also the amount consumed at night is relevant for acute glycemic control (45). Similarly, studies in controlled laboratory settings show that restricting food intake to the daytime during night work can prevent the development of glucose intolerance and misalignment of glucose rhythms (45). We hypothesized that aligning meal timing and composition better to the internal clock, would improve glucose tolerance and insulin sensitivity. A plausible explanation for the absence of detectable changes in our postprandial glucose and insulin response is that the nutrition intervention, which was only followed during night shifts, was not long enough to structurally alter glucose and insulin sensitivity. A human laboratory study showed that eating at night increased postprandial glucose area under the curve following meal tolerance tests, comparing to not eating at night, after 4 days of simulated night work (46). The meal composition in our study was based on work by Gupta et al. (16), which primarily aimed to reduce short-term effects of night eating (e.g., subjective hunger, gastric discomfort, and sleepiness during night shifts). Therefore, it seems that the timing of food intake has more impact on postprandial glucose and insulin responses than the macronutrient composition. Insulin sensitivity and endogenous glucose production are highly sensitive to food acting as a zeitgeber, which may play a role in the effects of meal timing interventions on endogenous circadian glucose rhythms (47).

Despite the absence of changes in postprandial glucose and insulin responses to the standardized mixed-meal test, the nutrition intervention was associated with changes in several continuous glucose monitoring (CGM)-derived metrics during daily life, both on day shift and night shift days. Notably, GMI, HBGI and MGE increased, suggesting higher mean glucose exposure and more pronounced glucose excursions. These findings suggest a potentially unfavorable shift in certain aspects of glucose dynamics. However, this pattern was accompanied by a modest increase in time in range (TIR) and occurred in the absence of detectable deterioration in postprandial glucose and insulin responses derived from the mixed-meal challenge test. Therefore, the CGM findings do not consistently point towards worsening glucose metabolism.

One possible explanation is that advancing food intake altered the temporal distribution of glucose exposure across the day rather than overall glucose regulation. In this scenario, glucose excursions may have become more concentrated during specific periods while glucose values remained within the target range for a larger proportion of the day. Such a redistribution could explain the simultaneous increase in TIR and excursion-based metrics. Previous work suggests that glucose responses to standardized snacks vary across the day, although differences were observed between real-life versus laboratory settings (48). In a real-life setting among healthy adults, this study reported that the highest glucose excursions were observed in the morning, while more dampened but prolonged response was observed in the evening (48). As such, advancing intake earlier during the waking period may have contributed both to higher peak glucose responses, as well as to an increase in time spent within the target range. In contrast, the personalized sleep intervention was associated with decreases in TIR, GMI, MGE and HBGI despite no targeted changes in meal timing. This suggests that sleep-related factors may influence glucose dynamics differently from meal-timing interventions. Earlier research indicated indeed associations between sleep behavior with CGM-derived glucose patterns (49). In addition, a modest increase in diet quality (DHDI), observed specifically in the sleep intervention group, may have contributed to these changes in CGM-metrics. No comparable changes were observed in the control group, indicating that the CGM-metrics remained relatively stable in the absence of intervention. However, given the self-selected study design and baseline differences between groups, these observations should be interpreted descriptively and not as evidence of differential intervention effects. Taken together, these findings suggest that advancing the timing of food intake influenced the distribution of glucose levels across the day rather than overall glucose metabolism. In the absence of changes in postprandial glucose and insulin responses, these shifts are unlikely to reflect a meaningful improvement in metabolic health. Given the exploratory nature of the CGM analyses and the relatively small magnitude of the observed changes, larger studies with detailed temporal analyses of CGM profiles are needed to determine whether these changes represent a clinically meaningful adverse effect or a redistribution of glucose responses across the day.

### 4.1 Strengths and limitations

The most important strength of this study is that the interventions were implemented in a real-life occupational setting. While laboratory studies have reported beneficial effects of altered meal timing and dietary composition on biological and cognitive outcomes (16, 25), This study extends those findings by evaluating cardiometabolic effects under real-world conditions. Another strength is the use of a standardized mixed-meal tolerance test to assess cardiometabolic and inflammatory health, which is a sensitive methodology for detecting the health effects of a relatively mild nutritional intervention (40). This study has shown that applying personalized meal plans is feasible in this particular work setting. The homogeneity of the shift work roster further strengthened the study by allowing more reliable comparison across groups and occasions. In addition, the inclusion of data from the sleep intervention and control group, where available, supported interpretations of the findings by showing that the advanced meal timing was specific for the nutrition intervention.

This study also has several limitations. First, all participants were male, which limits generalizability to the broader shift working populations. Second, dietary intake was self-reported, which may have resulted in underreporting of energy intake and inaccuracies in portion size estimation and macronutrient content calculations (50). Third, postprandial challenge data were only available for the nutrition intervention group due to practical considerations. Consequently, changes in postprandial lipid, glucose, insulin, and inflammatory responses could only be evaluated within the nutrition intervention group. The absence of comparable measurements in the sleep intervention and control groups precludes causal attribution of these changes to the nutrition intervention and does not allow exclusion of alternative explanations such as temporal trends, seasonal influences, or regression to the mean. In addition, participant self-selection resulted in baseline differences between study arms, precluding causal interpretation of between-group differences.

### 4.2 Implications and future directions

This study investigated the effects of a personalized nutrition intervention focused on meal timing and macronutrient distribution in night shift workers with a relatively unfavorable cardiometabolic risk profile as compared to age-matched men in the general population, including a high prevalence of elevated total cholesterol (68% vs. 53%) and elevated fasting glucose (30% vs. 10%) (51, 52). This underscores the need for feasible interventions in this shift work population. Taken together, these findings suggest that a relatively modest intervention, focused on rearranging timing and macronutrient composition of the main meals around and during night shifts, was feasible, even in a high-demanding workplace. Despite the fact that the intervention was only followed around night shifts, it still resulted in overall advanced timing of food intake and beneficial alterations in postprandial lipid and inflammatory responses. Greater awareness of the importance of meal timing could be an effective strategy in promoting health of shift workers. There has been increased attention within several work sectors for the health risks of night shift work. Our nutrition intervention could be one of the strategies to apply in the workplace to enhance cardiometabolic health. Although the effects in our study were small, effects may become more relevant when applied for longer (years) periods of time. Future studies could assess whether similar interventions yield larger effects on glucose metabolism in populations with impaired metabolic health, such as individuals with type 2 diabetes, or when combined with energy restriction.

## 5 Conclusion

A 3-month personalized nutrition intervention in night shift workers advanced the timing of energy intake without altering caloric intake or diet quality. This shift in meal timing was associated with lower postprandial lipid and inflammatory response patterns within the nutrition intervention group. Continuous glucose monitoring-derived metrics showed modest changes, suggesting alterations in glucose dynamics without improvements in overall glucose control. Together, these findings indicate that modifying meal timing and macronutrient distribution may support cardiometabolic health in shift workers in a real-world occupational setting.

## Data Availability

The raw data supporting the conclusions of this article will be made available by the corresponding author, without undue reservation.
