# Supplementary material for: A personalized nutrition intervention advances meal timing and is associated with reduced postprandial lipid and inflammatory responses in night shift workers
Source: Front Nutr. 2026 Oct 8;13:1912094. doi: 10.3389/fnut.2026.1912094 (PMC13687275; doi:10.3389/fnut.2026.1912094)
Supplement: Supplementary file 1 [file Table_1.DOCX]

**Supplementary Information S1.
Example of personalized feedback and nutrition advice to a participant.**
All participants received personal feedback and nutrition advice based on their personal dietary, cardiometabolic and continuous glucose monitoring data.

**TIMED Personalized Nutrition Advice for Participant 009**

The nutrition advice is developed using the following five steps:

1. Daily energy distribution across the 24-hour period
2. General macronutrient distribution per eating occasion
3. Personalized advice based on the PhenFlex test
4. Specific food recommendations based on CGM data and food diary records
5. Personal preferences and tailored advice based on laboratory results

**Step 1. 24-hour energy distribution.**

Estimated energy requirement (participant 009): **2576 to 2904 kcal/day**

**Recommended energy distribution:**

- **Breakfast:** 25% of total daily energy intake (in the morning, potentially after the night shift and before going to sleep)
- **Afternoon snack:** 15% of total daily energy intake (after waking from daytime sleep)
- **Early dinner:** 40% of total daily energy intake (between approximately 18:00 and 20:00)
- **Light night meal:** 20% of total daily energy intake (between approximately 00:00 and 02:00)

Based on food diary records from **Specified dates** (two night shifts and the day following the night shifts).

Other

End shift-sleeping

Awake-18:00

**Stap 2. General Macronutrient Distribution per Eating Occasion**

Macronutrient distribution from food diary (participant 009) overlaid with recommended macronutrient distribution per eating occasion:

End shift-sleeping

Awake-18:00

Protein

Fat

Carbohydrates

Other

**Recommended Macronutrient Distribution**

**Breakfast**

- 75% carbohydrate
- 15% protein
- 10% fat

**Afternoon snack**

- 25% carbohydrate
- 25% protein
- 50% fat

**Early dinner**

- 45% carbohydrate
- 20% protein
- 35% fat

**Light night meal**

- 55% carbohydrate
- 15% protein
- 30% fat

**Recommended Energy Intake per Eating Occasion**

| calculated EE: | 2576-2904 |  |  |  |
| --- | --- | --- | --- | --- |
| **GRIP 009** | **kcal** | **Carbohydrates (kcal)** | **Fat  (kcal)** | **Protein (kcal)** |
| breakfast (25%) | 726 | 545 | 73 | 109 |
| Afternoon snack (15%) | 436 | 109 | 218 | 109 |
| Early dinner (40%) | 1162 | 523 | 407 | 232 |
| Night meal (20%) | 581 | 319 | 174 | 87 |

**Step 3. Dietary Advice Based on Insulin Resistance Subtyping**

Subtype: **Healthy**

**Step 4. Continuous Glucose Monitoring (CGM)**

Please refer to the corresponding CGM graphs for detailed glucose patterns and specific food related responses.

**Step 5: Personal Preferences and Laboratory-Based Recommendations**

- Coffee, cola, energy drinks (e.g., Red Bull), and caffeinated tea: Avoid caffeine during the 4 hours before sleep.
- Limit fluid intake during the 2 hours before sleep.
- Alcohol: Evaluate and avoid using alcohol as a sleep aid.

Fasting insulin: 40 pmol/L (target < 70 pmol/L)

Fasting triglycerides: 0,82 mmol/L (target <2,3 mmol/l)

Fasting total cholesterol: 4,4 mmol/L (target < 5 mmol/l)

**Nutrition Advice During and Around Night Shifts**

- Breakfast (25%) after nightshift
  - High carbohydrate meal: 75% carbohydrates, 15% protein, 10% fat
  - Option 1: 80 g oatmeal with 200 mL milk, 1 cup of blue berries, 30g of raisins
  - Option 2: 200 g Greek yogurt with 90 g granola and 1 banana
- Afternoon snack (15%) after daytime sleep
  - Higher-fat meal: 25% carbohydrates, 25% protein, 50% fat
  - Option 1: 3 wholgrain crispbreads, 50 g avocado, 55 g light cream cheese (8% fat), 6 slices chicken breast deli meat
  - Option 2: 15 g unsalted mixed nuts, 3 rice cakes, 20 g peanut butter
- Evening Meal (40%)
  - Early dinner: 45% carbohydrates, 20% protein, 35% fat
  - Option 1: 120 g brown rice (uncooked weight), 1 can natural tuna, 2 tbsp olive oil, 70 g corn, 200 g broccoli, 1.5 tomatoes, 10 g crispy fried onions, pinch of black pepper
  - Option 2: 125 g wholegrain penne (uncooked weight), 40 g Parmesan cheese, 2 tbsp olive oil, 100 g broad beans (frozen), 50 g green peas (frozen), 150 g green asparagus, pinch of black pepper
  - Option 3: 110 g pearl couscous, 100 g chicken thigh fillet, 1/2 zucchini, 1/2 eggplant, 1.5 tbsp olive oil, 1/2 onion, 1 bell pepper, 25 g unsalted almonds, 1 pinch of black pepper
  - Option 4: 300 g potatoes, 200 g green beans, 1 chicken burger, 3 tbsp olive oil, 125 gr Greek yogurt 2%, 1 cup blue berries
- Midnight snack (20%) during nightshift
  - 55% carbohydrates, 15% protein, 30% fat
  - Option 1: 3 slices wholegrain bread, 3 slices semi-mature cheese 48+, 1 banana
  - Option 2: 3 slices wholegrain bread, 25 g 100% peanut butter, 100 g banana
  - Option 3: 100 g full-fat quark, 100 gr banana, 120 g peach (frozen), 50 g oatmeal, 1 tbsp chia seeds

**Supplemental Table 1.** Weighted median time of energy intake (in minutes) for macronutrients and total caloric intake for all groups. Est; Estimate, CL; confidence limit, degrees of freedom 195. P-values are based on within group comparisons.

| **Outcome** | **Pairwise contrast  (vs baseline)** | **Group** | **Est** | **SE** | **Lower.CL** | **Upper.CL** | **t.ratio** | **p-value** |
| --- | --- | --- | --- | --- | --- | --- | --- | --- |
| Carbohydrate | Start | Nutrition | -125 | 48 | -220 | -30 | -2,60 | 0,010 |
|  | End | Nutrition | -79 | 32 | -142 | -16 | -2,45 | 0,015 |
|  | Start | Sleep | -104 | 58 | -219 | 10 | -1,80 | 0,074 |
|  | End | Sleep | -40,7 | 47 | -134 | 53 | -0,86 | 0,391 |
|  | Start | Control | 105 | 106 | -105 | 315 | 0,99 | 0,325 |
|  | End | Control | 142 | 103 | -61 | 345 | 1,38 | 0,170 |
| Fat | Start | Nutrition | -125 | 50 | -224 | -27 | -2,51 | 0,013 |
|  | End | Nutrition | -83,9 | 33 | -149 | -19 | -2,56 | 0,011 |
|  | Start | Sleep | -77,6 | 51 | -177 | 22 | -1,54 | 0,126 |
|  | End | Sleep | -36,6 | 49 | -133 | 60 | -0,75 | 0,455 |
|  | Start | Control | 105 | 110 | -112 | 321 | 0,96 | 0,341 |
|  | End | Control | 154 | 103 | -49 | 357 | 1,49 | 0,137 |
| Protein | Start | Nutrition | -129 | 49 | -226 | -32 | -2,63 | 0,009 |
|  | End | Nutrition | -84,1 | 33 | -149 | -20 | -2,57 | 0,011 |
|  | Start | Sleep | -70,9 | 49 | -168 | 27 | -1,43 | 0,153 |
|  | End | Sleep | -36,5 | 49 | -133 | 60 | -0,75 | 0,456 |
|  | Start | Control | 111 | 109 | -104 | 327 | 1,02 | 0,310 |
|  | End | Control | 149 | 104 | -57 | 354 | 1,43 | 0,154 |
| Total | Start | Nutrition | -150 | 48 | -246 | -55 | -3,10 | 0,002 |
|  | End | Nutrition | -87,5 | 30 | -146 | -29 | -2,95 | 0,004 |
|  | Start | Sleep | -68,3 | 48 | -163 | 27 | -1,42 | 0,158 |
|  | End | Sleep | -33,3 | 50 | -131 | 64 | -0,67 | 0,502 |
|  | Start | Control | 128 | 109 | -87 | 343 | 1,17 | 0,242 |
|  | End | Control | 172 | 105 | -35 | 378 | 1,64 | 0,103 |

**Supplemental Table 2.** Total caloric intake for all groups over time. Estimates are in kcal (Standard error). P-values are based on within group comparisons.

| Group | Total caloric intake | | | P-value | |
| --- | --- | --- | --- | --- | --- |
|  | **Baseline (run-in)** | **Start Intervention** | **End intervention** | **Start vs baseline** | **End vs baseline** |
| Nutrition | 1965 (156) | 1856 (208) | 1988 (186) | 0,47 | 0,90 |
| Sleep | 2159 (618) | 1870 (202) | 1670 (227) | 0,66 | 0,49 |
| Control | 1703 (302) | 1604 (287) | 1817 (299) | 0,73 | 0,73 |
